# Supplementary material for: A drug repurposing screen reveals dopamine signaling as a candidate therapeutic pathway for PIGA-CDG
Source: bioRxiv. 2026 Apr 18:2026.04.17.719256. Preprint. [Version 1] doi: 10.64898/2026.04.17.719256 (PMC13105143; doi:10.64898/2026.04.17.719256)
Supplement: Supplement 1 [file media-1.pdf]

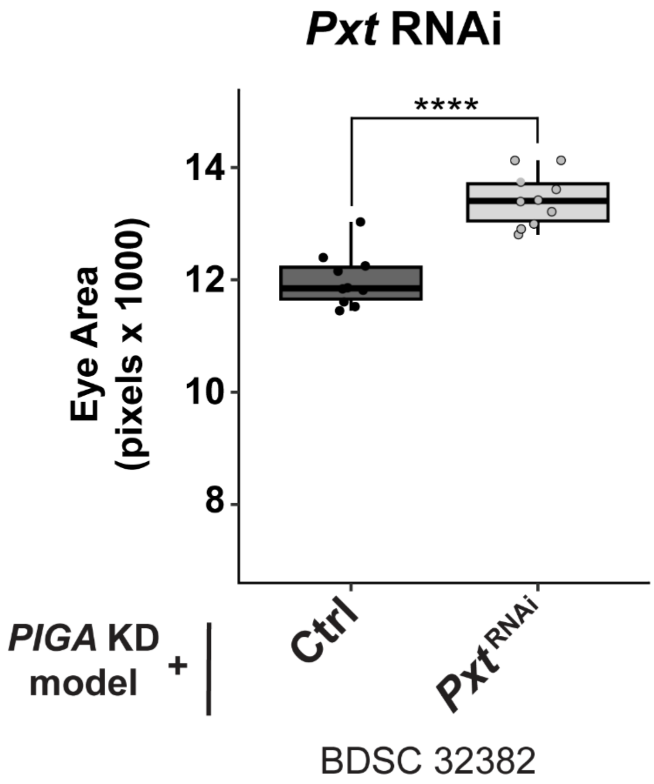

S1 Fig: Secondary RNAi validation for Pxt. RNAi against COX-like gene *Pxt* (BDSC 32382) partially rescues eye size of *PIGA* eye model. Statistical significance was determined using unpaired two-tailed t-test. **** p<0.0001.
