# Supplementary material for: A drug repurposing screen reveals dopamine signaling as a candidate therapeutic pathway for PIGA-CDG
Source: bioRxiv. 2026 Apr 18:2026.04.17.719256. Preprint. [Version 1] doi: 10.64898/2026.04.17.719256 (PMC13105143; doi:10.64898/2026.04.17.719256)
Supplement: Supplement 3 [file media-3.pdf]

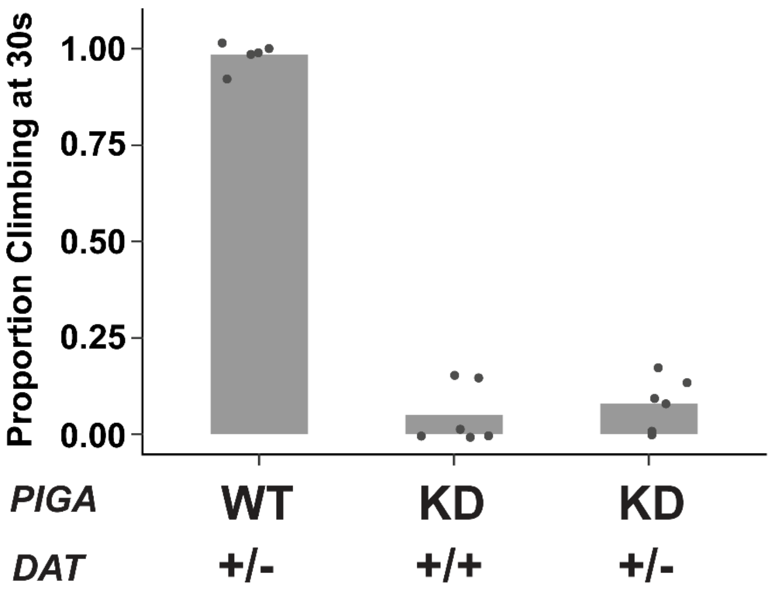

S3 Fig. *DAT* loss does not impact pan-neuronal *PIGA* knockdown climbing phenotype. Heterozygous loss of *DAT* improves the severe climbing impairment in pan-neuronal *PIGA* knockdown female flies. Only females were tested, as males were too severely impaired to be properly assessed.
